# Influence of anaesthetic factors on skin graft viability in a burns ICU

**DOI:** 10.1186/cc14439

**Published:** 2015-03-16

**Authors:** C Isitt, KA McCloskey, A Cabello, P Sharma, MP Vizcaychipi

**Affiliations:** 1 Chelsea and Westminster Hospital, London, UK

## Introduction

Graft failure is a major cause of morbidity in patients with burns, resulting in increased length of hospital stay and increased number of operations. At our regional burns unit we collated the data from anaesthetic charts of patients admitted to our burns ICU who required skin grafting. The aim was to analyse whether any anaesthetic variables contribute to graft failure.

## Methods

Thirty-five patients were included in the analysis with a total of 191 operations. These were a combination of debridement, split skin grafts (SSG) and change of dressings. All patients were admitted to our burns ICU between January 2009 and October 2013. Exclusion criteria were death prior to discharge and initial surgery at a different hospital. Sixteen patients had good graft viability (Group A) and 19 patients had poor graft viability (Group B). Logistical regression was performed using SPSS (Version 22.0). Hosmer and Lemeshow testing was used to confirm goodness of fit. Independent variables were age, sex, preoperative haemoglobin, intraoperative fluid resuscitation, blood products, inotropes, volatile agents and temperature. Poor graft viability was defined as requiring at least one additional skin graft. Analysis was performed on all operations and then by subtype of operation (that is, SSG and debridement, SSG only).

## Results

There was no significant difference in age, %total burn surface area or Belgian Outcome Burns Injury score between the groups. For all operation data, use of colloids was found to significantly contribute towards poor graft viability (*P *= 0.035, 95% CI). When analysis was performed on only SSG and debridement operations, colloids remained significant (*P *= 0.034, 95% CI) and metarminol use was found to significantly contribute (*P *= 0.028, 95% CI) to poor graft viability. Overall use of inotropes was not significant between the two groups. Other variables including minimum and maximum temperature, preoperative haemoglobin and blood transfusion were not found to be significant.

## Conclusion

Our tesults suggest that the use of colloids is a contributor to poor graft viability in burns. This was found to be independent of temperature and overall inotrope use; however, the use of metarminol may be a contributing factor.

